# TRP channels: Role in neurodegenerative diseases and therapeutic targets

**DOI:** 10.1016/j.heliyon.2023.e16910

**Published:** 2023-06-02

**Authors:** Mashoque Ahmad Rather, Andleeb Khan, Lianchun Wang, Sadaf Jahan, Muneeb U. Rehman, Hafiz A. Makeen, Syam Mohan

**Affiliations:** a Department of Molecular Pharmacology & Physiology, Bryd Alzheimer's Research Institute, Morsani College of Medicine, University of South Florida, Tampa, United States; b Department of Pharmacology and Toxicology, College of Pharmacy, Jazan University, Jazan 45142, Saudi Arabia; c Department of Medical Laboratory Sciences, College of Applied Medical Sciences, Majmaah University, Al Majma'ah, 11952, Saudi Arabia; d Department of Clinical Pharmacy, College of Pharmacy, King Saud University, Riyadh, 11451, Saudi Arabia; e Pharmacy Practice Research Unit, Department of Pharmacy Practice, College of Pharmacy, Jazan University, 45142, Saudi Arabia; f Substance Abuse and Toxicology Research Centre, Jazan University, Jazan, Saudi Arabia; g School of Health Sciences, University of Petroleum and Energy Studies, Dehradun, Uttarakhand, India; h Center for Transdisciplinary Research, Department of Pharmacology, Saveetha Dental College, Saveetha Institute of Medical and Technical Science, Saveetha University, Chennai, India

**Keywords:** TRP, Ca^2+^ homeostasis, Alzheimer's disease, Parkinson's disease, Huntington's disease, Amyotrophic lateral sclerosis

## Abstract

TRP (Transient receptor potential) channels are integral membrane proteins consisting of a superfamily of cation channels that allow permeability of both monovalent and divalent cations. TRP channels are subdivided into six subfamilies: TRPC, TRPV, TRPM, TRPP, TRPML, and TRPA, and are expressed in almost every cell and tissue. TRPs play an instrumental role in the regulation of various physiological processes. TRP channels are extensively represented in brain tissues and are present in both prokaryotes and eukaryotes, exhibiting responses to several mechanisms, including physical, chemical, and thermal stimuli. TRP channels are involved in the perturbation of Ca^2+^ homeostasis in intracellular calcium stores, both in neuronal and non-neuronal cells, and its discrepancy leads to several neuronal disorders such as Alzheimer's disease (AD), Parkinson's disease (PD), Huntington's disease (HD), and Amyotrophic lateral sclerosis (ALS). TRPs participate in neurite outgrowth, receptor signaling, and excitotoxic cell death in the central nervous system. Understanding the mechanism of TRP channels in neurodegenerative diseases may extend to developing novel therapies. Thus, this review articulates TRP channels' physiological and pathological role in exploring new therapeutic interventions in neurodegenerative diseases.

## 1 Introduction

Neurodegenerative diseases (NDs) are the principal cause of cognitive dysfunction and globally affect millions of elder populations and are extensively associated with progressive functional loss of neurons in specific brain regions, causing memory impairment and motor neuron dysfunction [1,2]. The pathogenesis implicates abnormal aggregation and accumulation of specific proteins as intracellular or extracellular deposits that are distinctive for each disease. Various factors, including genetic and environmental, cause the acquisition and misfolding of proteins [3,4]. NDs are incurable and divergent in their pathophysiology, with some triggering cognitive impairments and others aggravating a person's motor functions.

The TRP gene was first discovered in Drosophila and has since been found to be a large family of proteins expressed in both invertebrates and vertebrates [5,6]. TRP proteins are cation channels that are found in most cell membranes and respond critically to changes in the environment. The TRP family comprises 28 mammalian cation channels, which are subdivided into six subfamilies: TRPC (canonical), TRPV (vanilloid), TRPM (melastatin), TRPA (ankyrin), TRPML (mucolipin), and TRPP (polycystic) [7].

The TRPC sub-family contains seven homologs that are receptor-operated channels and are involved in intracellular calcium homeostasis. The TRPV subfamily comprises TRPV1-TRPV6 members, which can be activated by chemical and thermal stimuli and participate in osmolarity, thermo-sensing, and renal calcium absorption or reabsorption [7]. The TRPM subgroup contains eight mammalian members and has isoforms in most eukaryotic organisms. The members of this group have specific ion selectivity, and their gating and regulatory mechanisms are customized to integrate multiple signaling pathways.

TRP ion channels participate in various physiological and pathological conditions in electrically excitable and non-excitable cells by regulating magnesium influx and mediating the direct influx of calcium ions. TRPA1 initially thought to be a noxious cold sensor, is now considered a chemo-nociceptor and a promising analgesic target [8]. It is expressed in both peptidergic and non-peptidergic neurons, as well as certain myelinated Aβ-fibers and non-neuronal cells, including epithelial cells, mast cells, melanocytes, and fibroblasts [9].

TRPML channels are the smallest in the TRP superfamily, mostly localized to intracellular compartments, and are involved in a group of vesicular trafficking events [10]. The TRPML1 channel is abundantly expressed and displayed in signal transduction, membrane trafficking, and late endosomes and lysosomes ion homeostasis [11]. It concedes permeability to monovalent and divalent cations, including Na^+^, Fe^2+^, K^+^, and Ca^2+^.

The TRPP (transient receptor potential polycystin) subfamily consists of integral membrane proteins and can be divided into TRPP1 and TRPP2 proteins. This subfamily is considered the earliest member of the TRP family due to its presence in both prokaryotes and eukaryotes [12]. TRPP2 is homologous to other TRP channels, but TRPP1 does not have much resemblance and is a nonfunctional ion channel; therefore, it was redesignated as Polycystin1 (PC1). PC1 is an integral membrane glycoprotein, highly expressed and widely distributed in the kidney, pancreas, liver, and brain tubular epithelial cells. PC1 glycoprotein forms a signaling complex with the TRPP2 proteins, which plays a significant physiological role from maintaining left-right symmetry to tubular morphogenesis.

TRP channels are broadly manifested in human organs like the brain, kidneys, heart, and lungs and exhibit permeability to cations of metal ions, playing a substantial role in cell homeostasis, neurogenesis, and structural and functional plasticity [13,14]. TRP channels can be stimulated by an array of gated mechanisms, including voltage and ligand binding, covalent modifications of nucleophilic residues, and thermal changes Various ion channels, including TRPs in the brain and TRP subfamilies expressed in neurons and microglia, are implicated in the extension of neurodegenerative diseases and neuropathic pain [15]. TRPs are involved in the assimilation of inflammatory mediators linked to neurotoxicity or neuroprotection, where they support intracellular calcium regulation.

This review outlines the role of TRPs in the normal physiology of neurons and the disruption of Ca^2+^ homeostasis, providing an overview of the underlying mechanism of TRPs in the pathogenesis and regulation of several neurodegenerative diseases. This understanding of TRPs' role may lead to the development of novel therapeutic approaches.

## 2 Role of Ca^2+^ in the pathogenesis of ND

Calcium ions (Ca^2+^) play a crucial role in directing several neuronal functions and act as second messengers in various cellular processes. The regulation of intracellular Ca^2+^ ion concentration is essential for maintaining cellular functions smoothly. Even a slight increase in its concentration can lead to the activation of several cellular processes, including muscle contraction, neuronal transmission, and gene transcription. Upregulation of intracellular Ca^2+^ and elevated Ca^2+^ influx via voltage-dependent Ca^2+^ channels have been reported to cause age-related alterations and activate neurons. Sustained intracellular Ca^2+^ disturbances are immediate causes of neurodegenerative diseases (NDs) [16]. The diverse functions of neurons are dependent on Ca^2+^ signaling, which is influenced by the influx of Ca^2+^ from the extracellular environment or the release of Ca^2+^ from intracellular stocks in the endoplasmic reticulum (ER). The concentration of Ca^2+^ ions in the cytosol is relatively low at the resting stage but shows a gradual increase after activation [17].

The Ca^2+^ level in the ER is higher than in the cytosol at the resting stage. After activation, Ca^2+^ is discharged from the ER via two types of channels, the inositol-1,4,5-triphosphate receptors (IP3R) and the ryanodine receptors (RyR). Through RyR release, Ca^2+^ is triggered by a slight extension in cytosolic Ca^2+^ levels, and absolution through IP3R demands inositol-1,4,5-triphosphate (IP3). IP3 is executed by phospholipase-C from phosphatidylinositol-4,5-bisphosphate, which is promoted by G-protein coupled receptors in the plasma membrane. The influx of Ca^2+^ into the cytosol is triggered by the decline of ER calcium stores via store-operated Ca^2+^ channels stationed in the plasma membrane.

In NDs, the expulsion of Ca^2+^ is decreased via the plasma membrane Ca^2+^ pump, which leads to instability in Ca^2+^ homeostasis. Both ER and mitochondria are closely associated with Ca^2+^ homeostasis, in which ER participates in Ca^2+^ signaling, and mitochondria prevent excessive cytosolic Ca^2+^ load. Ca^2+^ channels actively participate in neuronal functions, and their signaling equates to membrane excitability, which is essential for short-term and long-term synaptic plasticity. The mitochondria are involved in the formation of cytosolic Ca^2+^ signals, in which the mitochondrial Ca^2+^ uniporter ion channel promotes the rapid and immense influx of calcium into the mitochondria [18]. Neurons are sensitive to changes in intracellular Ca^2+^ concentration, and even subtle defects and deregulation of Ca^2+^ signaling can have destructive consequences.

One possible significant ion influx pathway might be the instigation of TRPM channels. TRPM7 channels are perceptive to Ca^2+^ and Mg^2+^ and are efficiently adjusted by intracellular Mg^2+^ levels, which are prominent features in various NDs [[19], [20], [21], [22], [23], [24], [25], [26], [27]].

In conclusion, Ca^2+^ plays a critical role in neuronal functions, and instability in Ca^2+^ homeostasis is involved in NDs due to the high dependence on Ca^2+^ signaling to influence the function of neurons. Various ion channels in the plasma membrane compactly synchronize the mobilization of cations. Understanding the role of Ca^2+^ in the pathogenesis of NDs may help develop effective treatments for these diseases.

## 3 The TRP superfamily: role in the normal physiology of neurons

TRP ion channels are widely distributed in the brain and are localized in the hippocampus, cortex, cerebellum, thalamus, amygdala, substantia nigra, and striatum [26,27]. TRPs balance neurite outgrowth and axonal pathfinding in the immature hippocampus. Ca^2+^ acts as an intracellular precursor to reveal information and regulate neuronal function, and its signaling plays a crucial role in regulating physiological processes in neurons, including growth, survival, and differentiation. In Ca^2+^-dependent physiological events, neurons involve several modes of Ca^2+^ influx through plasma membrane channels. TRPC channels are actively involved in neuronal development, proliferation, and differentiation and play a vital role in attenuating synaptic plasticity, long-term potentiation, and neurosecretion. Implementing various pharmacological agents in neuronal cells leads to upregulating intracellular Ca^2+^ ions [28]. Its higher level is imputed after the release of Ca^2+^ from intracellular ER stores and influx across the membrane through TRPC channels.

Moreover, the entry of Ca^2+^, followed by ER store-reduction, attains decisive cellular functions. Ca^2+^ entry stocks ER stores, as it has a limited capacity, thus, making it competent to release Ca^2+^ upon subsequent stimuli, and its concentrations in ER must be maintained to carry out various fundamental functions. Depletion of Ca^2+^ in the ER could affect trafficking and protein folding and obstruct cellular functions reliant on Ca^2+^ influx. It is revealed that Ca^2+^ levels are crucial for regulating gene expression, muscle contraction, neurosecretion, the inclusion of electrical signaling, synaptic plasticity, differentiation of neurons, and apoptosis-mediated degeneration of neurons. Even though across the plasma membrane, entry of Ca^2+^ is controlled by several mechanisms like store depletion or membrane potential alterations, which in turn activates voltage-gated Ca^2+^ channels.

TRPC channels represent a sub-group of the ion channels, which act as non-selective Ca^2+^ entry channels with a distinct way of activation [29]. The entry of Ca^2+^ via the G-protein coupled receptors actively participates in synaptic transmission, action potentials, and sensory transduction [30,31], and alterations in Ca^2+^ influx regulate various cellular structures, including the axonal cones and dendritic filopodia of evolving neurons [32]. Therefore, TRPC channels may be important in attenuating these essential neuronal operations. It is documented that activation of TRPC1 modulates proliferation in neuronal stem cells, and TRPC3 and TRPC6 are intimated with BDNF-mediated neuronal growth [33,34]. The alterations can explain these defined physiological implications in specificity and functional activation of individual TRPC channels in multiple neuronal populations. Table 1 provides detail of the family of TRP channels having different roles in the normal functioning of neurons and in disease conditions.

**Table 1:** Classification of TRP channels.

TRP Channels		Function	Distribution in Brain regions	Disorders	References
TRPV Subfamily	TRPV1	Enhance neuronal death by agonist, such as Capsaicin, pain and noxious thermal sensing	Hippocampus, cortex, cerebellum, olfactory bulb, amygdala, mesencephalon and hindbrain	AD, PD	[101]
	TRPV4	Stimulates and upregulates neuronal inflammatory responses Prevent release of pro-inflammatory cytokine, thermal sensing	Hippocampus, cortex, cerebellum and thalamus	AD	[102]
TRPM Subfamily	TRPM2	Activate due to ROS, apoptotic cell death, Ca^2+^ entry in pancreatic beta cells, temperature dependent,	Hippocampus, cortical neurons, substantia nigra and striatum	ALS, PD, AD,	[103]
	TRPM7	Aggravate cell damage by increase Ca^2+^ induced oxidative stress, cell cycle regulation, Mg^2+^ homeostasis, entry to trace metals, promote cell growth and survival	Mouse cortical neurons Hippocampus, cerebrum, cerebellum and truncus encephali	ALS, AD,	[104,105]
TRPC Subfamily	TRPC1	Attenuates neurotoxicity and unfolded protein response, Regulate SOCE and amplifies survival of dopaminergic neuron, coupling to glutamate receptors, facilitate slow excitatory postsynaptic currents	Hippocampus, amygdala, cerebellum, substantia nigra and inferior colliculus	ALS, PD,	[106]
	TRPC3	Arbitrate neuronal differentiation, vasomotor function, inhibit to release cytokines and NO	Widely distributed in rat CNS Human dopaminergic neurons Globus pallidus striatum, Cerebellum	AD, ALS, PD	[107]
	TRPC4	Activate growth of neurite length, vasomotor function, microvascular permeability	Mouse hippocampal pyramidal neurons Frontal cortex, lateral septum, and ventral subiculum, Amygdala	Anxiety, Depression	[80]
	TRPC5	Reduce elevation of SOCE, brain development, growth cone morphology and guidance,	Hippocampus, Frontal cortex, Cerebellum, substantia nigra, Amygdala, Striatum, hypothalamus	ALS, PD	[106]
	TRPA1	Activation and progression of astrocytes, facilitates Ca^2+^ entry, promote activation of proinflammatory cytokines	Hippocampus, Brain stem, cerebral cortex	AD, Migraine	[108,109]
TRPP Subfamily	TRPP1	Acts as mechanoreceptor, sensing external stress, triggering cascade of signaling pathways	Endothelial cells lining BBB, ER membranes	Psoriasis, Muscular dystrophy, AD, PD, Renal hypoplasia	[110]
	TRPP2				
	TRPP3		Hippocampus, Cerebellum, olfactory bulb, thalamus, midbrain		

## 4 Role of TRP channels in various neurodegenerative diseases

### 4.1 Alzheimer's disease

Alzheimer's disease (AD) is a devastating neurodegenerative disorder characterized by severe memory loss and behavioral changes. The underlying pathology involves the accumulation of extracellular amyloid aggregates called senile plaques and intracellular neurofibrillary tangles, leading to the selective loss of synapses and neurons in the hippocampal and cerebral cortical regions [35]. While the amyloid and tau hypotheses have been the traditional theories explaining AD, the dysregulation of calcium homeostasis has recently gained attention as a critical factor in AD pathogenesis [36].

Calcium is an essential intracellular messenger that binds to multiple proteins, receptors, and ion channels to regulate various physiological functions. During neurodegeneration, neurons become inefficient in regulating calcium levels. Enhanced AD pathological lesions induce neurotoxicity and cytokines, leading to dysregulation of calcium homeostasis and leaving neurons prone to excitotoxicity and apoptosis [37].

The failure of calcium homeostasis is a prerequisite for developing neurons, synaptic plasticity, and metabolic pathways, and its alteration plays a crucial role in regulating neuronal death in AD. Aβ aggregation may induce ER calcium release into the cytosol, resulting in a calcium cytosolic burden, which reduces glutathione levels, leading to intracellular reactive oxygen species (ROS) generation and accumulation [38,39]. Additionally, Aβ deposition induces microglial activation, and the subsequent proinflammatory cytokines promote neuronal damage and death [40]. TRPV1 channels also promote neuroinflammatory processes, while capsaicin activation of TRPV1 channels protects the hippocampus against Aβ peptide pathology [[41], [42], [43], [44]].

TRPA1 channels are essential in the progression of non-neuronal cells such as astrocytes. Aβ pathology stimulates TRPA1-dependent calcium entry, astrocyte activation, and transcription factor such as NF-кB to promote proinflammatory expression [45,46]. Deficient TRPM2 channels triggered by Aβ42 peptides avert TNF-α production and microglial activation, highlighting the critical function of TRPM2 channels in microglial activation [47,48].

Microglia and astrocytes activated by Aβ produce TNFα, which induces TRPM2 activation, and NO, which selectively modifies Cys553 and Cys558 residues to activate TRPC5. Ca^2+^ influx via nitrosylated TRPC5 mediates Ca^2+^-dependent NO production by neuronal NOS [49].

**Fig. 1:**
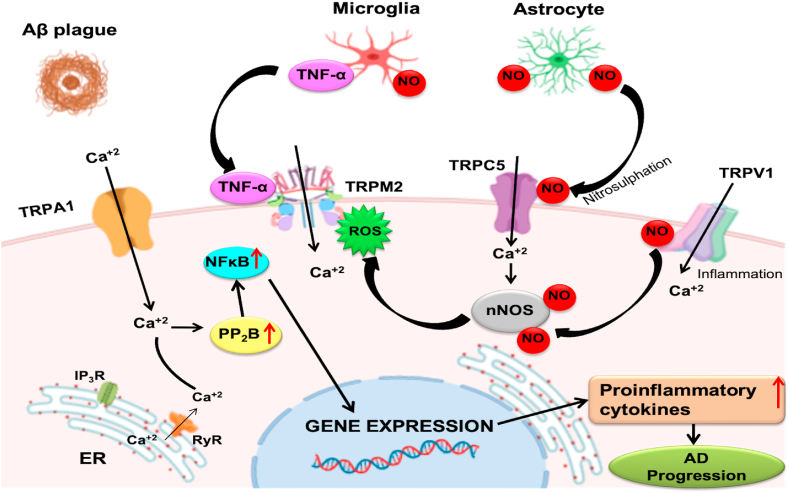
Role of TRP channels in Alzheimer's disease. Aβ-peptide stimulates the TRPA1 and enhances the ROS generation, which destabilizes the intracellular Ca2+ homeostasis, and initiation of IP3R initiates ER Ca2+ store depletion, resulting in the elevation of cytoplasmic Ca2+ which increases the expression of PP2B and NFкB. Generation of ROS, released in the cytosol, can trigger TRPM2 and a subsequent increase in intracellular Ca2+ ions induce NO production. Activated astrocytes and microglial cells by Aβ promotes TNFα, activating TRPM2 and NO, which stimulates TRPC5 and mediates Ca2+-dependent NO production by neuronal NOS. Aβ triggers the activation of neuroinflammatory processes, directed by activated-glial cells that produce inflammatory cytokines, which activate TRPV1. All these events cause neuronal death and eventually lead to the pathogenesis of AD.

Moreover, antioxidants such as glutathione regulate free radicals, and their supplementation significantly reduces TRPM2 expression during aging. Thus, reduced levels of antioxidants, cytokines, and Aβ production in AD stimulate several TRP channels that can increase intracellular calcium and induce excitotoxicity and apoptosis [50]. [Fig. 1].

Understanding the role of calcium dysregulation and TRP channels in AD pathology is critical for developing potential therapeutic strategies for this debilitating disease. Recent research has suggested that novel treatment modalities will be explored to treat human TRP channel-based diseases in the future [50].

### 4.2 Parkinson's disease

Parkinson's disease (PD) is the second most predominant neurodegenerative disorder related to aging after AD. It is a movement disorder, depicted by the disintegration of dopaminergic neurons (DNs) in the substantia nigra (SN), that worsens over time. Lewy bodies are the cytoplasmic inclusions formed by α-synuclein protein and are considered the main pathological hallmark of PD. Several factors in PD can lead to the loss of DNs in SN, such as oxidative stress, mitochondrial dysfunction, protein aggregation, and alteration in calcium homeostasis [51,52]. Studies reported that TRP channels could facilitate some of the mechanisms that advance the progression of the disease. A study on SHSY5Y neuroblastoma cells demonstrated that expression of TRPC1 proteins diminished while incubating with neurotoxin Salsolinol and was detected in cerebrospinal fluid and nigrostriatal cells of PD patients [53]. Salsolinol may lead to the translocation of TRPC1 from the cell membrane into the cytosol, as its expression was increased in the cytosol than in the cell membrane [54]. It is evidenced that NMSAL, a derivative of Salsolinol, was found to be more noxious, perceived in the nigrostriatal and intraventricular fluid samples of PD patients, and displayed identical events to those of Salsolinol in the expression and localization of TRPC1, which specifies a defensive role for TRPC1 in PD. Studies manifested that MPTP infusion in mice attenuates the levels of TRPC1 in the SNpc.

**Fig. 2:**
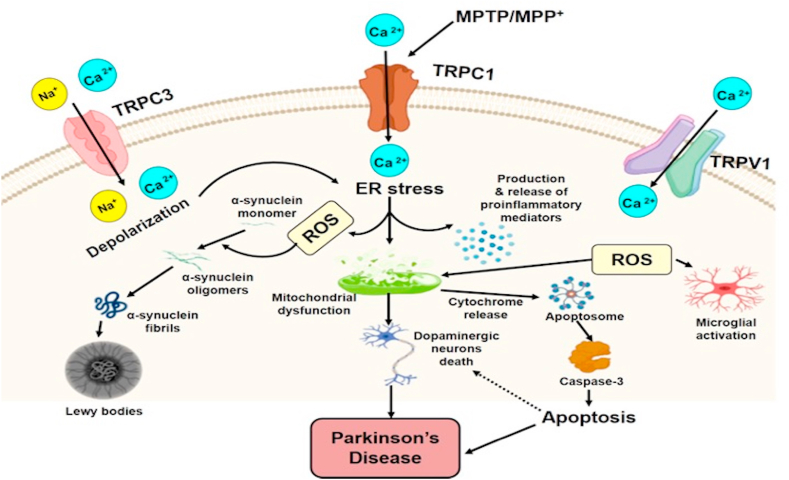
Role of TRP channels in Parkinson's disease. PD causes the stimulation of TRPV1, TRPC1, and TRPC3 channels, as well as upregulates ROS formation. Initiation of TRPC1 triggers mitochondrial dysfunction. Inhibition of TRPV1 results in PD and upregulates ROS formation and inflammatory process. Initiation of TRP channels augments the intracellular Ca2+ levels, inflammatory response, and mitochondrial dysfunction, which eventually triggers the apoptotic cascade activation and neuronal loss in PD.

Similarly, MPP + reduces its expression in PC12 cells [54,55]. In contrast, the overexpression of TRPC1 proteins advances the PC12 cell survival rate by preventing mitochondrial membrane depolarization and leading to the expression of antiapoptotic Bcl2 and Bcl-xl genes. TRPC1 is indispensable in protecting DNs against toxicity produced by the activated TRPV1 channel. Moreover, TRPV1 activation causes upregulation of intracellular Ca^2+^, leading to cytochrome-c release, caspase-3 cleavage, and mitochondrial disruption [54]. DNs rely on the inception of Na+ and Ca2+ channels, and MPTP neurotoxin enhances the Ca2+ activity by diminishing the expression of TRPC1, leading to Ca^2+^ liberation into the cytosol. Overexpression of TRPC1 downregulates caspase-3, suppresses MPP^+^-caused cell death, and enables Ca^2+^ interaction, which is important for the consistency of DNs in PD [56]. MPP^+^ neurotoxin directly activates microglia and promotes the extension of several proinflammatory agents [57]. Activated microglia trigger the release of proinflammatory cytokines and chemokines that induces the DNs to decline, manifesting glia-mediated toxicity to these neurons. TRPC3 overexpression upregulates Ca^2+^ levels, which supplements the nitric oxide suppression induced by BDNF-activated microglia [58]. TRPC3 in the SN GABA projections synchronizes the redundancy of these neurons and retains the constant Na^+^ influx that gives rise to depolarized potential [59]. However, ROS-affected TRPC3 activity trigger more depolarized potential in GABAergic neurons [59]. There was the activation of TRPM2 in primary hippocampal neurons after MPP treatment [60]. The group described that the MPP-treated hippocampal neurons activate oxidative stress-induced TRPM2 channels causing apoptotic death pathways [60]. In microglia, it was reported by another group that glutathione depletion with MPP-induced TRPM2 channel activation causes oxidative toxicity [15]. [Fig. 2].

### 4.3 Huntington's disease

Huntington's disease (HD) is an autosomal neurodegenerative disorder triggered by polyglutamine expansion in Huntingtin protein and represented by cognitive impairment, medium spiny neurons (MSN) loss in the striatum, and convulsive movements [61]. Various channels have been reported to alter the K^+^ homeostasis, such as the Kir4.1 channel expressing striatal astrocytes in mutated HTT protein that disintegrates the extracellular K^+^ homeostasis hence provokes hyperexcitability in neurons, i.e., HD motor symptoms in striatal neurons. However, the normal Kir4.1 channel is a prevalent astrocytic K^+^ channel that plays a prominent role in balancing the cells resting membrane potential and buffering K^+^ ions in the brain [62]. Furthermore, it is suggested that HD mHTT protein modifies the high voltage stimulated Ca^2+^ channels [63]. Besides dysfunction in the Ca^2+^ channel, other ion channels have also exhibited reduced expression in several HD mouse models. Thus, modification in these ion channels disintegrates the ion homeostasis in cortical pyramidal neurons, due to which synaptic integration, neurotransmitter release, and genetic expression gets affected, which plays a central role in cortical dysfunction in HD.

**Fig. 3:**
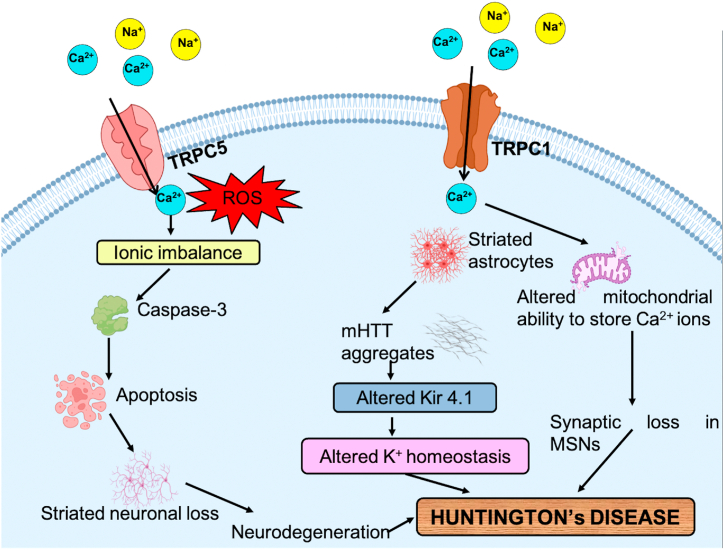
Role of TRP channels in Huntington's disease. HD leads to the stimulation of TRPC1 and TRPC5 channels and increases the generation of ROS via the influx of cations into the cell. Kir4.1 channel alters the K+ homeostasis and activates striatal astrocytes in mHTT protein, which causes hyperexcitability in neurons. Alteration in Ca2+ homeostasis leads to mitochondrial dysfunction and loss of synapsis in MSNs. TRPC1 and TRPC5 account for the excess influx of Ca2+ ions involved in Ca2+-dependent apoptosis in the striatum, which causes striatal neuronal loss and ultimately leads to HD.

The Ca^2+^ homeostasis and signaling perturbation were marked in most of the HD models, and the genes that encode TRPC6 and IP3R1 were found to be upregulated, and other genes involved were downregulated in Ca^2+^ signaling [64,65]. A study has revealed that RNAi-mediated knockout of TRPC1 and TRPC6 reclaims YAC128 MSN spines and repressed impulsive SOCE in these spines, whereas the knockout of other TRPC genes didn't retrieve spine loss in YAC128 MSNs. Palmitate is an unsaturated fatty acid abundantly found in the brain, and the process by which it gets anchored to the membrane is called palmitoylation. This lipid modification process increases the hydrophobicity of proteins in neuronal receptors and ion channels, providing a stronger alliance with membrane lipids, therefore affecting the structure, function, trafficking, and overall stability of protein [[66], [67], [68]]. In HD, palmitoylation assists in reinstating neuronal function by decreasing the activity of caspase-6 [67]. Several studies have demonstrated that S-palmitoylation in TRP channels regulates many life cycle phases of ion channels [68,69] and the central role of TRPML3 palmitoylation in autophagy [70]. Activation of GPCR stimulates TRPC5 channels, and these channels congregate as homo and heterotetrameric systems with TRPC1 and TRPC4 subchannels and are constantly functional [71,72]. It is proposed that S-glutathionylation at Cys-176 and Cys-178 of TRPC5 accounts for additional Ca^2+^ influx and enhanced Ca^2+^-dependent apoptosis in the striatum of HD [73,74]. Therefore, depalmitoylation of pathological TRPC5 could avert enhanced Ca^2+^ deposition with inefficient plasma membrane localization. [Fig. 3].

### 4.4 Amyotrophic lateral sclerosis

Amyotrophic lateral sclerosis (ALS) is a neurological disorder characterized by the progressive loss of motor neurons in the brain stem, motor cortex, and spinal cord, resulting in muscle weakness, atrophy, and contraction. The disease is associated with the hyperexcitability of axons due to the continuous conduction of sodium ions (Na^+^) and subsequent reduction in potassium ion (K^+^) conduction [75,76]. The lingual muscles, stimulated by motor neurons, are also susceptible to degeneration in ALS, linked to the differential expression of voltage-gated calcium channels (VGCCs).

Studies have shown that superoxide dismutase 1 (SOD1) blocks the mitochondrial voltage-dependent anion channel-1 (VDAC1) and promotes mitochondrial-dependent apoptosis, leading to fatal paralysis in ALS. The transient receptor potential melastatin 7 (TRPM7) ion channel is widely distributed in most cellular tissues, providing a pathway for the influx of Ca^2+^, Mg^2+^, and trace metal ions [77]. Mg^2+^ ions are involved in cell viability and proliferation, and alterations in their homeostasis due to changes in TRPM7 expression can contribute to pathological conditions such as ALS [78].

**Fig. 4:**
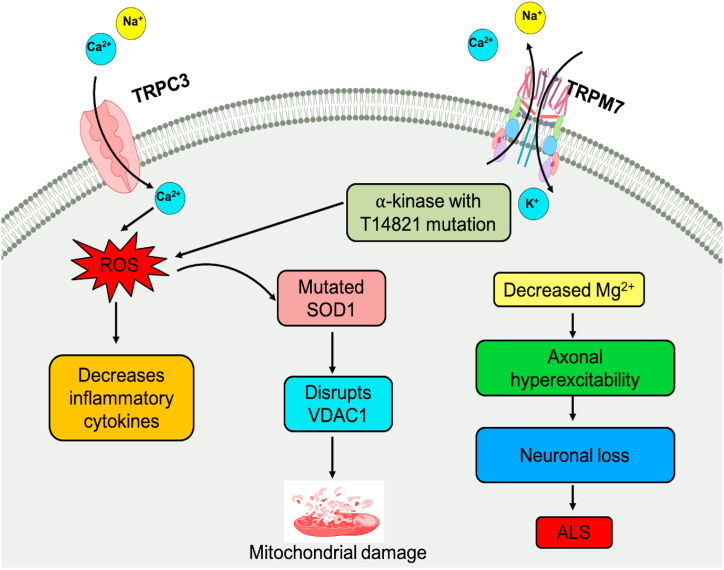
Role of TRP channels in Amyotrophic lateral sclerosis. In ALS, activation of voltage-gated Na + ion channels and decreased conduction of K+ ions causes hyperexcitability of axons. ROS generation causes SOD1 dysfunction, which disrupts the VDAC1 and causes mitochondrial-dependent apoptosis, and alteration in the Mg2+ ion homeostasis contributes to the etiology of ALS.

In ALS patients, a TRPM7 channel variant, T14821, has been detected that localizes between the channel and the kinase region [79]. Although this variant does not show significant changes in α-kinase activity, it exhibits increased susceptibility to suppression by intracellular Mg^2+^ within the physiological range. Inhibition of TRPM7 could worsen Mg^2+^ homeostasis in low Mg^2+^ conditions, reducing intracellular Mg^2+^ content and contributing to the complications of the NDs [78,79]. [Fig. 4].

### 4.5 Other neurodegenerative diseases (vascular cognitive impairment, spinocerebellar ataxia, spinal muscular atrophy)

Vascular cognitive impairment (VCI) is caused by the interruption of blood flow or damage to the blood vessels within the brain, leading to irreparable neuronal damage. It is evidenced that TRPA1 channels play a significant role in cerebral blood flow regulation. TRPA1 proteins are present on perivascular nerves, and their initiation leads to vasodilation via the liberation of C-protein gene-associated peptides [80]. In this neurological dysfunction, the oxygen level decreases when the brain encounters a shortage of blood supply, causing the generation of free radicals, which later bind to other molecules in the cells and make them dysfunctional. TRPA1 protein channel forms the inner lining of blood vessels, makes a channel that allows calcium ion signaling when activated, and forms wider arteries in the brain. A study has revealed that free radicals are the agents behind the activation of TRPA1 proteins, resulting in the vasodilation of blood vessels [81], indicating that this protein channel plays an essential role in protecting vascular blood supply and brain damage.

Spinocerebellar ataxia (SCA) is an autosomal inheritance neurodegenerative disorder in which the cerebellum and other connected regions of the brain start to degenerate. It is considered that TRP channels could promote some processes that lead to the progression of the disease. A study on moonwalker mice has revealed that mutation in TRPC3 protein leads to abnormal channel opening and passage of Purkinje cells and cerebellar ataxia. This model observed impaired growth and development of Purkinje cell dendritic spindles. Thus, TRPC3 proteins play an essential role in both progression and continuity of dendritic and Purkinje cells intervening in cerebellar ataxia [82]. Therefore, TRPC channels mediate the degeneration of neurons on the excessive influx of calcium ions and regulate neuronal development [83].

Spinal muscular atrophy (SMA) is an autosomal neuromuscular disease depicted by the disintegration of alpha motor neurons in the spinal cord, which directs advancing proximal muscle fragility and paralysis. Studies have implicated that TRPV4 gene mutation has been connected to three apparent axonal neuropathies, including Scapuloperoneal spinal muscle atrophy (SPS MA), Congenital distal spinal muscle atrophy (CDSMA), and Charcot Marie Tooth disease type 2C (CMT2C). TRPV4 gene mutation causes CDSMA atrophies, SPSMA, and HMSN IIC, affecting channel maturation and leading to decreased expression of functional TRPV4 channels [84]. It has been shown that TRPV4 mutation enhances channel movement, modifies Ca2+ homeostasis and peripheral neuropathies, and is a putative treatment option for these disorders [85]. Mutation in TRPV4 promotes toxicity to cells and enhances integral and functional channel current, which can activate the deterioration of the peripheral nerves. It was documented that TRPV4 mutants have a physiological localization and exhibit the augmented activity of the Ca^2+^ channel [86,87]. Therefore, TRPV4 increased function mutations lead to a more intracellular influx of Ca^2+^ and emerge to trigger the etiology of TRPV4-associated axonal neuropathies.

## 5 Targeting TRP channels for therapy of diseases: a new hope

TRP proteins comprise a group of cation channels found in most cell membranes and are explicated in the plasma membrane, helping to attenuate the thrust for the entry of Na^+^, Ca^2+^, K^+^, Mg^2+^, and trace metal ions [88]. Deposition of Aβ peptides induces activation of microglial cells and proinflammatory cytokines [41], which provokes TRPV1 channels to promote neuroinflammatory processes. TRPV1 proteins also get activated by capsaicin induction which protects the hippocampus against Aβ pathology. TRPA1 channels actively participate in the progression of astrocytes, and its activation is caused by Aβ peptides which stimulate TRPA1-dependent Ca^2+^ entry. TRPM2 channels play an essential role in the instigation of microglial cells and TNF-α production [47]. Moreover, GSH antioxidant also significantly diminishes TRPM2 expression. Thus, reduced levels of antioxidant expression and Aβ production in AD stimulate several TRP channels.

Various factors can cause the loss of DNs; one such factor is TRP channels that could facilitate and promote the disease's progression. TRPV1 channel arouses death of mesencephalic DNs, and activation of TRPV1 leads to Ca^2+^-dependent cell death [89]. In addition, the TRPV1 channel increases the intracellular levels of Ca^2+^ that lead to mitochondrial disruption [55]. Toxicity caused by the activated TRPV1 channel is aborted by the TRPC1 channel protecting DNs against toxicity. In HD, various channels alter the K^+^ homeostasis in mutant HTT protein, such as the Kir4.1 channel causes hyperexcitability in HD motor neurons by disintegrating the extracellular K^+^ homeostasis. Under normal conditions, the Kir4.1 channel plays a vital role in balancing the resting potential of the cells [59]. The gene codes for TRPC6, and IP3R1 were more expressed, and other genes were less expressed in Ca^2+^ signaling [61]. TRPC5 proteins promoted the extra entry of Ca^2+^ and enhanced Ca^2+^-dependent apoptosis in the striatum of HD [90], thus preventing excessive deposition of Ca^2+^.

In ALS neurological disorder, the perpetual conduction of Na^+^ ions and subsequent decline in K^+^ ion conduction are liable for the hyperexcitability of axons [75]. Extensive distribution of TRPM7 channel in cells and tissues equipping influx of Ca^2+^, Mg^2+^, and other metal ions [76]. TRPM7 channel causes the alteration in the homeostasis of Mg^2+^ ions, which leads to pathological consequences such as ALS, and the inhibition of the TRPM7 channel could worsen the Mg^2+^ homeostasis in Mg^2+^ deficient environment, the diminishing volume of intracellular Mg^2+^ ions, consequently contributes to the pathology of the NDs [80]. TRP channels are part of cellular pathways that evoke the integration of several inflammatory markers linked with neuroprotection or neurotoxicity, where they synchronize intracellular calcium and signaling [16,17]. Thus, TRP channels receive more attention as feasible targets for the therapy of NDs.

Based on the results, TRP channel regulations play a promising role in Ca^2+^-dependent neuronal death in NDs. TRPA1 is reflected as a chemo-nociceptor and acts as the best target for analgesics [90]. TRPC channels have been instrumental in synchronizing neurosecretion, long-term potentiation, and synaptic plasticity and function as non-selective Ca^2+^ influx channels [3]. TRPC1 plays a significant role in the neurotoxicity of DNs proteins and regulates neural stem cell proliferation. TRPC3 and TRPC6 are allied with BDNF-mediated neuronal growth [91], and TRPC3 channels synchronize the redundancy of neurons in the SN and retain constant Na^+^ influx, which generates a toxic depolarized potential [59]. TRPC5 activity gets attenuated by depalmitoylation and exhibits satisfying effects against oxidative stress by downregulating toxicity caused by TRPC5 [89]. TRPV1 channels promote neuroinflammatory responses and protect the hippocampus against Aβ toxicity [45]. TRPML channels reside in intracellular compartments and play an essential role in vesicular trafficking events [8], and TRPML1 is involved in signal transduction, membrane trafficking, and ion homeostasis [9]. TRPM2 ion channels prevent the activation and production of microglia and TNF-α, thus playing a pivotal role in microglial activation [48].

TRPM8 might have a cholesterol-mediated role in neuronal cell dynamics which can be a potential target for neurodegenerative diseases.

TRPM7 equips an ion channel pathway influx of Ca^2+^, Mg^2+^, and other metal ions, and its activation is dependent on intracellular Mg^2+^ levels, which emerges as a key factor in various NDs and the regulation of TRP channels can be a novel therapeutic option for NDs.

MicroRNAs (miRNAs) are crucial regulators of gene expression and play a vital role in biological processes, including the pathologies of the human system. Studies have identified numerous miRNAs associated with the progression of neurodegenerative diseases (NDs), making them a potential therapeutic option for these diseases. It is estimated that miRNAs regulate one-third of human genes involved in essential cellular processes and associated pathological events [92].

Different miRNAs, such as miR-9, miR-79, miR-124, miR-132, miR-134, and miR-137, play a crucial role in neuronal development and synaptic plasticity [93,94]. Dysregulation of miRNAs can trigger neuronal deficits, leading to the advancement of NDs. Therefore, altering the content of miRNAs may prove beneficial in the disease pathologies associated with NDs [95]. Several studies have reported that miRNA dysregulation influences disease pathogenicity, supporting their involvement as a novel therapeutic option to prevent disease progression [96].

In experimental studies, increasing the expression of miRNA-16 via an external source of an osmotic pump attenuated the expression of Allograft inflammatory factor 1 (Aif1) and Glial fibrillary acidic protein (Gfap), promoting neuronal protection and protection from oxidative damage. Moreover, enhancing the content of miR-16 via cell transfection in an in-vitro model resulted in the attenuation of Tau phosphorylation and the expression of BACE1 and APP genes [97]. Infusing miR-132 into the mouse model triggered the enhanced contents of inositol 1,4,5-trisphosphate 3-kinase B, associated with Aβ accumulation and Tau phosphorylation [98].

Studies have investigated the potential role of miRNAs in regulating the expression of the LRRK2 gene, resulting in the protection of deficient neurite outgrowth [99]. In another study, miR-124 regulated BIM protein to attenuate apoptosis and lysosomal destruction, confirming its neuroprotection via regulating the translocation of proapoptotic protein Bax [100]. Additionally, miRNA plays an essential role in regulating the expression of the mHTT gene, which was demonstrated in a study on transgenic mice models and HD iPSCs, resulting in the blockage of mHTT and proving to be a therapeutic option for preventing the disease [101].

In conclusion, miRNAs play a critical role in regulating gene expression and are associated with the progression of neurodegenerative diseases. Dysregulation of miRNAs can trigger neuronal deficits, leading to the advancement of NDs. Altering the content of miRNAs may prove beneficial in preventing disease progression, making them a potential therapeutic option for neurodegenerative diseases.

## 6 Conclusion

It is widely recognized that TRP ion channels play a crucial role in the normal functioning of the nervous system, and their dysfunction has been implicated in the pathogenesis of several neurodegenerative disorders (NDs). Aberrations in TRP channel function can be attributed to various pathological processes that disrupt brain homeostasis, such as oxidative stress, mitochondrial dysfunction, inflammation, and protein misfolding. These processes can alter the permeability and selectivity of ion channels, leading to changes in neuronal excitability, synaptic plasticity, and, ultimately, neurodegeneration.

Therefore, understanding the mechanistic processes underlying the involvement of TRP channels in NDs could help in the development of novel therapeutic strategies for these disorders. Recent studies have highlighted the potential of TRP channels as therapeutic targets for NDs, as their increased activity has been observed in several neurodegenerative conditions. Modulation of TRP channel activity has been shown to have neuroprotective effects and improve cognitive function in preclinical models of NDs.

In conclusion, the involvement of TRP channels in NDs has emerged as an exciting area of research with significant implications for developing effective treatments for these devastating disorders. Further studies are needed to unravel the complex mechanisms underlying the association between TRP channels and NDs and to identify specific TRP channel subtypes as potential therapeutic targets.

## Production notes

### Author contribution statement

All authors listed have significantly contributed to the development and the writing of this article.

### Data availability statement

Data included in article/supp. material/referenced in article.

## Declaration of competing interest

The authors declare the following financial interests/personal relationships which may be considered as potential competing interests:One of the author Dr Syam Mohan is a member of Editorial board.
